# A review on xylanase sources, classification, mode of action, fermentation processes, and applications as a promising biocatalyst

**DOI:** 10.5114/bta.2024.141806

**Published:** 2024-09-30

**Authors:** Tariku Abena, Addis Simachew

**Affiliations:** 1 Microbial Biotechnology Research Program, National Agricultural Biotechnology Research Center (NABRC), Ethiopian Institute of Agricultural Research, Addis Ababa, Ethiopia; 2 Institute of Biotechnology, Addis Ababa University, Addis Ababa, Ethiopia

**Keywords:** biocatalyst, fermentation, mode of action, xylanase

## Abstract

The utilization of hydrolytic enzymes in various industrial processes worldwide has gained more attention than chemical catalysts due to the high selectivity of enzymes, their ease of control, and their negligible environmental impact, as they produce very small amounts of byproducts. Xylanase is one such enzyme that catalyzes the breakdown of the β-1,4 linkage of xylan, the second most abundant renewable heteropolysaccharide and hemicellulosic constituent of the plant cell wall. Naturally, xylanase can be obtained from various sources such as mollusks, insects, plants, animals, and various microorganisms (bacteria, fungi, yeast, and algae). The utilization of xylanase could greatly improve the overall economics of processing lignocellulosic materials for the generation of monosaccharides, liquid fuels, and chemicals. Microbial xylanase is suitable for applications in food and feed, paper and pulp, textile, pharmaceutical, and biorefining industries. It has gained global attention due to its substrate specificities, biochemical properties, and various biotechnological applications. This review focuses on xylanase production, sources, fermentation processes, modes of action, purification methods, and applications in various industries.

## Introduction

Lignocellulosic biomass has been recognized as a sustainable source of renewable energy and value-added products such as animal feed, single-cell protein, ethanol, xylitol, organic acids, methane, and monosaccharides. However, converting lignocellulosic waste into these value-added products faces difficulties, including low selectivity and high cost (Malhotra and Chapadgaonkar, 2018). Hemicelluloses consist of heterogeneous groups of polysaccharides existing in an irregular form and located between the cellulose fibrils and lignins (Peng et al., 2012). The components of hemicellulose include various sugars, such as the five-carbon sugars or pentose sugars (xylose, rhamnose, and arabinose), six-carbon sugars or hexoses (glucose, mannose, and galactose), and uronic acids (4-*o*-methyl glucuronic, D-glucuronic, and D-galacturonic acids) (Prasad and Sethi, 2013; Tarasow et al., 2013; Pandey et al., 2014). The homopolymer or heteropolymer backbone of hemicellulose is mostly linked by β-1,4-glycosidic bonds and occasionally by β-1,3-glycosidic bonds (Bajpai, 2016). To hydrolyze hemicelluloses into their constituent sugars, chemical or enzymatic methods can be employed (Branco et al., 2019). However, the utilization of chemical methods is not cost-effective and not environmentally friendly compared to enzymatic methods (Howard et al., 2003).

Xylan, the dominant hemicellulose in the plant cell wall, is a hetero-polysaccharide integrated into the lignocellulosic structure, consisting of β-1,4 linked D-xylosyl residues, and accounts for about 33% of all renewable organic biomass (Girio et al., 2010; Motta et al., 2013; Liu et al., 2015). Xylan has many applications that directly or indirectly affect human life. For example, arabinoxylans, a type of xylan found in rye and wheat, influence the processing, quality, and nutritional values of bread and the brewing properties of grains (Vinkx and Delcour, 1996). The corn fiber-bran arabinoxylans of starch have served as food additives, thickeners, and stabilizers, as well as film formers and emulsifiers (Doner and Hicks, 1999; Yadav et al., 2009). Xylan is a major constituent of lignocellulosic biomass for biofuel production (Carroll and Somerville, 2009) and animal feed (Bedford and Partridge, 2010). Lignocellulosic biomass from softwood and hardwood trees is most commonly used for construction, pulp and paper products, and biorefinery applications that involve separating the biomass into its individual components to produce various bio-products (Xavier et al., 2010; Pei et al., 2016; Zhu et al., 2016).

The existence of xylan in a heterogeneous and complex form makes its hydrolysis difficult; consequently, the complete breakdown of xylan into its components requires a large variety of enzymes (Subramaniyan and Prema, 2002). Due to the chemical nature of xylan, which is highly heterogeneous and complex, the complete breakdown of this biomass demands the action of different hydrolytic enzymes (Subramaniyan and Prema, 2002).

Endoxylanases and β-xylosidases (commonly referred to as xylanases) are the two major xylanolytic enzymes responsible for the hydrolysis of xylan into its constituents (Bajpai, 2022). Endoxylanases produce xylo-oligomers by breaking the homopolymeric backbone of 1,4-linked β-D-xylopyranose, whereas β-xylosidases act on xylooligomers to release xylose (Ahmed et al., 2009; Knob et al., 2010). Endoxylanases are particularly important as they are directly involved in the cleavage of glycosidic bonds, liberating short xylo-oligosaccharides (Verma and Satayanarayana, 2020). Biocatalysts have gained more attention compared to chemical catalysts due to their high specificity, ability to operate under mild pH and temperature conditions, low energy consumption, and positive environmental impact (Girelli et al., 2020). Xylanase enzymes have been utilized in various industries such as food, animal feed, pulp and paper, textiles, and biorefining (Pinheiro et al., 2021).

**Fig. 1:**
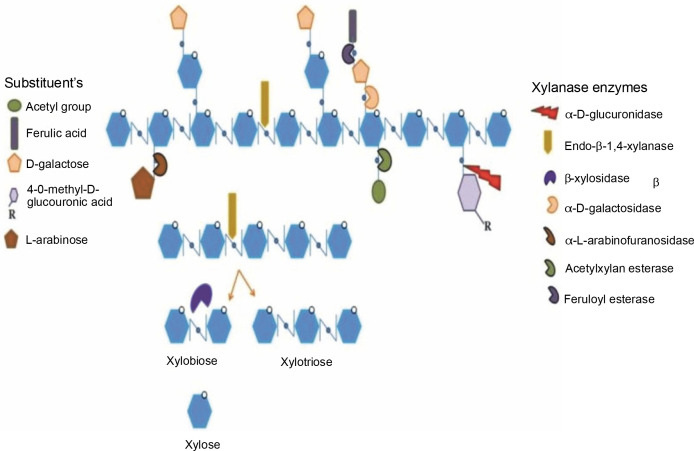
Schematic presentation of the structure of xylan with its bonds that are attacked by specific xylanolytic enzymes for the complete hydrolysis of xylan to its constituents. Adapted from the review paper by Bhardwaj et al. (2019)

Thus, this paper presents the classification of xylanase, its mode of action, different xylanase-producing sources, production methods, and purification methods, as well as its potential applications in different industries. The structure of xylan, with its bonds that are targeted by specific xylanolytic enzymes for the complete hydrolysis of xylan into its constituents, is presented in Figure 1.

## Mode of action of xylanase

Two different mechanisms, retention and inversion, are involved in the hydrolysis process of the xylan polymer by xylanase enzymes (Subramaniyan and Prema, 2002; Lombard et al., 2014).

### Retention

The retention process is a double displacement mechanism in which an α-glycosyl enzyme intermediate and an oxo-carbonium intermediate are formed, with glutamate residues acting as catalysts for hydrolysis. This double displacement mechanism for anomeric retention of the product was first proposed by Koshland (1953) and later reported by Clarke et al. (1993). Primarily, the two carboxylic acid residues present in the active site result in the formation of an α-glycosyl enzyme intermediate. This occurs through the protonation of the substrate by one carboxylic acid residue (acting as an acid catalyst), while another carboxylic acid residue causes a nucleophilic attack. This process collectively results in β to α inversion due to the formation of the α-glycosyl enzyme intermediate (Bhardwaj et al., 2019).

**Fig. 2:**
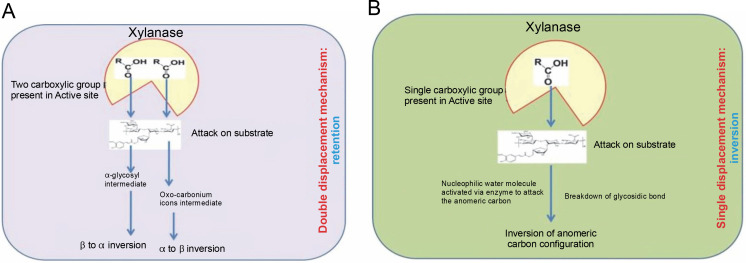
Mode of action of xylanase retention (A) and inversion (B); adapted from Bhardwaj et al. (2019)

In the second displacement, the anomeric carbon gives rise to the product with the β configuration (α to β inversion) through a transition state involving oxocarbonium ions (Collins et al., 2005; Lombard et al., 2014). Generally, the double displacement mechanism involves an acid catalyst that protonates the substrate, a carboxyl group of the enzyme, a covalent glycosyl enzyme intermediate with carboxylate, in which the anomeric configuration of the sugar appears opposite to that of the substrate, and oxo-carbonium ions that form covalent intermediates from both directions along with various noncovalent interactions (Clarke et al., 1993). The hydrolysis of xylan by the retention process is presented in Figure 2A.

### Inversion

Inversion is a single displacement mechanism, in which only one carboxylate ion offers for overall acidcatalyzed group departure. The enzymes that use this single displacement mechanism (inversion) are like enzymes families 8 and 43. These enzymes invert the anomeric center with glutamate and aspartate as the major catalytic residue and also act as the base for activating a nucleophilic water molecule to attack the anomeric carbon to cleave the glycosidic linkages and actualize inversion of anomeric carbon configuration (Collins et al., 2005; Motta et al., 2013; Lombard et al., 2014). Most of the polysaccharide hydrolyzing enzymes like cellulases, cellulohydrolases, and xylanases are known to hydrolyze their substrates with the retention of the C1 anomeric con guration (Gebler et al., 1992). The hydrolysis of xylan by the inversion mechanism process is presented in Figure 2B.

## Sources of xylanase

Inversion is a single displacement mechanism in which only one carboxylate ion facilitates the overall acid-catalyzed group departure. The enzymes that use this single displacement mechanism, such as those in families 8 and 43, invert the anomeric center with glutamate and aspartate as the major catalytic residues. These residues also act as the base to activate a nucleophilic water molecule, which then attacks the anomeric carbon to cleave the glycosidic linkages and invert the anomeric carbon configuration (Collins et al., 2005; Motta et al., 2013; Lombard et al., 2014). Most polysaccharide hydrolyzing enzymes, including cellulases, cellobiohydrolases, and xylanases, are known to hydrolyze their substrates with the retention of the C1 anomeric configuration (Gebler et al., 1992). The hydrolysis of xylan by the inversion mechanism is presented in Figure 2B.

### Fungal xylanases

Xylanase is produced by various living organisms, including archaea (Wainø and Ingvorsen, 2003), bacteria (Chakdar et al., 2016), fungi (Chadha et al., 2019; Singh et al., 2019), actinomycetes (Hunt et al., 2016), insects (Brennan et al., 2004), algae (Jensen et al., 2018), plants and immature seeds (Sizova et al., 2011), protozoa (Béra-Maillet et al., 2005), mollusks (Yamaura et al., 1997), and nematodes (Mitreva-Dautova et al., 2006). Xylanase-producing microorganisms inhabit diverse ecological niches such as marine and terrestrial habitats with decayed plant biomass, thermophilic and mesophilic environments, as well as the rumen of ruminants (Chadha et al., 2019; Singh et al., 2019).

There are many advantages that make microorganisms the preferred choice over plants and animals. These advantages include rapid multiplication in a short period, small space requirements, high potential in waste biomass biodegradation, production under controlled and closed systems, ease of handling, and low production costs, as inexpensive substrates are used as carbon sources (Mitreva-Dautova et al., 2006). Microbial xylanase also has advantages over conventional chemical catalysts, such as high catalytic activity, high specificity to its substrate, high yield of product, easy biodegradability, environmental friendliness, and economic viability (Gote, 2004). Bacteria and fungi are widely used for the industrial production of xylanase; therefore, this study focuses on fungal xylanase.

### Bacterial xylanases

Filamentous fungi are particularly interesting producers of xylanase from an industrial perspective, as they produce extracellular xylanases and other auxiliary enzymes involved in the degradation of xylan into its components. Furthermore, fungi produce higher amounts of xylanase than yeasts or bacteria (Li et al., 2022). The fungal genera commonly producing xylanase include *Aspergillus*, *Coriolus versicolor*, *Fusarium*, *Phanerochaete chrysosporium*, *Trichoderma*, *Pichia*, and *Penicillium* (Singh et al., 2019). The basidiomycete white-rot fungi (WRF) also produce extracellular xylanases that act on various hemicellulosic biomasses used as food sources (Buswell and Chang, 1994) and produce metabolites for the pharmaceutical, cosmetic, and food industries (Qinnghe et al., 2004).

These enzyme-producing white-rot basidiomycetes include *P. chrysosporium* (Castanares et al., 1995), *C. versicolor*, which produces a complex of xylanolytic enzymes (Abd El-Nasser et al., 1997), and *C. subvermispora* (de Souza-Cruz et al., 2004). Fungi are preferred over bacteria for xylanase production due to their potential to yield higher amounts of enzymes, produce several xylan-degrading auxiliary enzymes (Polizeli et al., 2005), and ability to be active at high and low temperatures acidic and alkaline pH levels, and high salt content (Pandya and Gupte, 2012; Bonughisantos et al., 2015).

## Classification of xylanase

Xylanases can be categorized into three types based on their molecular mass and isoelectric point (PI), crystal structure, and kinetic properties (Motta et al., 2013; Liu and Kokare, 2017). When classified based on molecular mass and isoelectric point, xylanase enzymes are grouped into two categories: (a) high-molecular weight with a low isoelectric (acidic) point (HMWLI) and (b) low-molecular weight with a high isoelectric (basic) point (LMWHI). However, this classification has a drawback as it cannot describe all xylanases since not all xylanases fall under the HMWLI or LMWHI categories (Collins et al., 2002; Basit et al., 2020).

To address this limitation, a more suitable system was developed, focusing on the primary structure (crystal) and comparison of the catalytic domains (Collins et al., 2005; Basit et al., 2020). For more curated information on the characteristics and classification of enzymes, the Carbohydrate-Active Enzyme (CAZy) database, which is involved in the breakdown, modification, and assembly of glycosidic bonds in carbohydrates and glycoconjugates, is appropriate. This database consists of genomic, sequence annotation, family classifications, structural (3D crystal), and functional (biochemical) information from publicly available resources such as the National Center for Biotechnology Information (NCBI) (Lombard et al., 2014; Basit et al., 2020). The CAZy database (http://www.cazy.org) classification categorizes the xylanases into glycoside hydrolase (GH) families that include: 5, 7, 8, 9, 10, 11, 12, 16, 26, 30, 43, 44, 51, and 62. Among these families the 16, 51, and 62 GH families have two catalytic domains with bifunctional properties, unlike the 5, 7, 8, 10, 11, and 43 GH families that have a single distinct catalytic domain (Collins et al., 2005).

## Fermentation process for xylanase production

The production of xylanase depends on the selected microorganism, the composition of the growth medium, physicochemical growth parameters, and the fermentation method used for enzyme production. The growth medium contributes 30–40% to the total cost of enzyme production (Mitreva-Dautova et al., 2006). Xylanase production can be affected by the type and concentration of medium components such as the carbon source, nitrogen source, micronutrients, and macronutrients.

Physicochemical growth parameters, such as inoculum size, temperature, pH, agitation/aeration, water content of the substrate, incubation period, trace elements, and vitamins, significantly influence xylanase production (Abhishek et al., 2017; Thomas et al., 2017). Xylanase production involves two types of fermentation processes: submerged fermentation and solid-state fermentation (Agnihotri et al., 2010; Motta et al., 2013).

### Xylanase production under submerged fermentation (SmF)

Submerged fermentation (submerged culture) uses liquid fermentation media in which the substrate is dissolved in water. Despite the limitations related to space, energy, and water requirements, submerged fermentation is widely used in industrial processes due to its higher production yield, lower risk and cost of contamination, and ease of monitoring the fermentation process (Jain et al., 2013). Additionally, submerged fermentation is favored due to the lack of equipment for enzyme production under solid-state fermentation (Subramaniyam and Vimala, 2012). Approximately 90% of the total xylanase production worldwide utilizes the submerged fermentation (SmF) process (Polizeli et al., 2005). The submerged fermentation of aerobic microorganisms is a well-known and widely used method for producing cellulase and xylanase. SmF is generally preferred when preparations require more purified enzymes (Garcia-Kirchner et al., 2002).

### Xylanase production under solid-state fermentation

Solid-state fermentation (SSF) is a fermentation process that involves a solid matrix without free-flowing water, serving as physical support and a source of nutrition for microbial growth (Pandey et al., 2000; Singhania et al., 2009). The SSF system offers many advantages over SmF, such as mimicking the natural habitat of the microorganism, reducing water activity to minimize microbial contamination, providing greater enzyme stability, requiring less energy, producing enzymes with higher specific activities, and simplifying downstream processing (Beg et al., 2001; Singhania et al., 2009; Jain et al., 2013). Additionally, SSF provides higher enzyme yields than submerged fermentation (Agnihotri et al., 2010).

The SSF process is especially suitable for the growth of fungi, as these organisms can grow at relatively low water activities, unlike bacteria and yeast, which cannot grow at low water activity (Corral and Villasenor-Ortega, 2006). The production of enzymes like xylanase by SSF from fungi has received special attention due to its economic and engineering advantages (Pandey et al., 1999). Several research reports indicate the suitability of solid-state fermentation over liquid-state conditions for high yields of xylanase enzyme (Nair et al., 2008; Roy et al., 2013; Singh et al., 2013). SSF processes utilize agricultural, forestry, and food processing residues and wastes such as sugar cane bagasse, wheat bran, wheat straw, corn cobs, rice bran, rice husks, sawdust, and other similar substrates for xylanase production (Pandey et al., 1999; Corral and Villasenor-Ortega, 2006).

Although SSF has tremendous advantages, there are limitations and challenges that negatively affect this process. These include different gradients (moisture, temperature, substrate concentration, and others), bioreactor design (static bed bioreactor), heat dissipation, mass transfer, and control of fermentative parameters (Khanahmadia et al., 2006; Singhania et al., 2009). Therefore, more research on design, modeling, operation, and scaling up is necessary to enable the employment of SSF processes involving bioreactors.

## Purification of xylanase

Enzymes are essential biomolecules with a wide range of applications in industrial and biomedical fields. Therefore, purification of these biomolecules is a crucial pre-requisite for their effectiveness in industrial applications. The purification of xylanases from different microorganisms began in 1982 (Walia et al., 2014). Conventional purification methods, such as ultrafiltration, ammonium sulfate precipitation, gel permeation chromatography, and ion exchange chromatography, are commonly used for xylanase enzyme purification (Irfan and Sayed, 2012; Kamble and Jadhav, 2012; Walia et al., 2014).

Several researchers rely on these methods for xylanase enzyme purification from different sources. For example, Li et al. (2010) used DEAE 52 column and CM Sepharose Fast Flow chromatography for the purification of xylanase produced by *Streptomyces rameus* L2001. Taibi and coworkers (2012) purified endo-xylanase enzyme with a molecular mass of 70 kDa from *Penicillium* using ammonium sulfate fractionation, gel filtration on BioGel P10, DEAE cellulose, and CM Sephadex chromatographies (Taibi et al., 2012). Lopez and Estrada (2014) purified xylanase from *Trichoderma reesei* using ammonium phosphate precipitation, followed by DEAE-Sepharose CL-6B and Ultrogel AcA 44 (LKB) chromatographies. Another research report details the purification of two xylanases, Xyl I, and Xyl II, from the *Trichoderma inhamatum* strain using ion exchange chromatography (diethylaminoethanol DEAE-Sepharose) and subsequent gel filtration chromatography (Sephadex G-75) (Silva et al., 2015). de Oliveira Simões and coworkers (2019) extracted crude enzyme from *Myceliophthora heterothallica* by precipitating with 96% ethanol and performed xylanase purification with two steps of chromatography: gel filtration (Sephadex® G-75) and anion-exchange (Resource™ Q) (de Oliveira Simões et al., 2019).

However, the conventional xylanase enzyme purification methods mentioned above have drawbacks, such as being highly time-consuming, not cost-effective, and yielding low protein quantities (Iqbal et al., 2016; Ramakrishnan et al., 2016). To overcome these problems, a single-step liquid–liquid fractionation technique (aqueous two-phase system) has been developed (Glyk et al., 2015). The advantages of the aqueous two-phase system (ATPS) purification technique over conventional purification techniques include low energy consumption with high yield, use of cheaper materials, higher resolution, and not affecting the nature of the protein (Glyk et al., 2015; Iqbal et al., 2016; Ramakrishnan et al., 2016). Due to these advantages, some researchers have used this technique for purification. Garai and Kumar (2013) used this technique for the purification of alkaline xylanase from *Aspergillus candidus*. Ng et al. (2018) purified xylanase from *B. subtilis* using an alcohol/salt ATPS, while Gómez-García et al. (2018) and Bhardwaj et al. (2019) used ATPS with a PEG/salt system for purification of xylanase from *Trichoderma harzianum* and *Aspergillus oryzae* LC1, respectively.

## Application of xylanase

Xylanase is employed in various industries, including the paper and pulp industry for bleaching pulp, the fruit juice industry for higher yield and quality products, the bakery industry for improving the texture of bread, and as an animal feed additive to improve nutritional value and reduce feed costs (Pasalari and Homaei, 2022).

### Animal feed industry

Xylanase plays a crucial role in enhancing the nutritional values of agricultural residues and animal feeds. As animal feed additives, xylanase enzymes improve feed nutritional value by solubilizing and degrading insoluble feed constituents (Goswami and Pathak, 2013). Several research findings demonstrate the application of xylanases in animal feed to enhance its nutritional value and increase the weight gain of animals. For instance, research by Zhang et al. (2014) on broilers fed a wheat-based diet supplemented with xylanase indicated that the supplementation degraded the arabinoxylan backbone into its monomers (arabinose and xylose) in the ileum, jejunum, and duodenum, enhancing nutrient digestibility by decreasing digesta viscosity.

Paloheimo and coworkers reported that the utilization of xylanase as feed additives showed improved weight gain and enhanced feed conversion ratio (Paloheimo et al., 2010). Additionally, a study by Passos et al. (2015) demonstrated that adding xylanase to a corn and soybean meal-based diet for growing pigs improved nutrient digestibility and reduced digesta viscosity.

### Paper and pulp industry

There are two major processes involved in the paper and pulp industry: pulping and bleaching. These processes generate organic and inorganic pollutants, including lignins, tannins, resins, and chlorine compounds, which are environmental pollutants (Chandra et al., 2011). Currently, chlorine dioxide is commonly used as a bleaching agent in paper and pulp industries. However, it forms organochlorine compounds when combined with organic matter, causing genetic and reproductive damage to living organisms (Bajpai, 2012; Sharma et al., 2014).

#### Bioleaching

Bleaching is the process of removing lignin from pulp using chemicals, gases, steam, enzymes, etc., to produce bright and white finished paper (Beg et al., 2001). Utilizing enzymes in the paper and pulp industries not only avoids the use of hazardous chemicals like chlorine in the bleaching process (Garg et al., 2011) but also addresses the limitations of mechanical pulping processes (Sharma et al., 2016). Conventional pulping strategies face challenges such as precipitation or re-precipitation of xylan on the fiber, entrapment of lignin, and variations in brightness (Mathur et al., 2005).

To overcome these problems associated with traditional pulping and bleaching strategies, eco-friendly treatments, specifically enzymatic treatments, have been used. Among these, enzymatic treatment using xylanase has gained significant attention in the pulp and paper industries (Golugiri et al., 2012; Ho and Jamila, 2014). Xylanase enhances the bleaching process by hydrolyzing the bond that covalently associates xylan with cellulose and lignin, causing the separation of cellulose and lignin (Yang et al., 2019). The application of xylanases improves pulp brightness, and the purity of dissolved pulp, removes metal cations, reduces overall paper cost, decreases the time consumption of the pulping process, improves the permeability of the fiber surface, restores bonding, and eliminates the need for chlorine (Nagar et al., 2013; Yang et al., 2019).

Several reports are indicating the potential application of xylanase enzymes in the paper and pulp industries. Sridevi et al. (2017) demonstrated that pretreatment of paper pulp with xylanase produced by *Trichoderma asperellum* resulted in a reduced kappa number, higher brightness, and the removal of chromophores and hydrophobic compounds. Nair et al. (2010) reported that the application of xylanase produced by *Aspergillus sydowii* SBS 45 on Kraft pulp increased the brightness of the pulp from 29.42 to 70.42% and reduced the kappa number from 15.93 to 1.61. Gautam and coworkers demonstrated that xylanase from white rot fungi, *Schizophyllum commune* ARC-11, resulted in a maximum decrease in kappa number (14.51%) and a 2.9% improvement in pulp brightness when applied to ethanol-soda pulp from *Eulaliopsis* (Gautam et al., 2018). Pretreatment of hardwood Kraft pulp with xylanase produced by *Paenibacillus campinasensis* BL11 increased brightness (by as much as 4.4 and 3.9%) and viscosity (by as much as 0.5 and 0.3 cP) of the pulp after full chlorine dioxide bleaching for untreated and oxygen-delignified hardwood Kraft pulp (Ko et al., 2010). Raj et al. (2018) suggested that xylanase produced on wheat bran from alkaliphilic *Bacillus licheniformis* used as a pretreatment of Kraft pulp showed a 19% reduction in kappa number compared to control pulp after 2 h of treatment. The scanning electron microscopy (SEM) and infrared spectroscopy (FTIR) analysis of xylanase-treated pulp revealed significant morphological and structural changes in pulp fibers.

### Textile industry

The textile industry comprises three main processes: desizing (to remove the size or adhesive substance), scouring (to remove the inhibitory material from desized fibers), and bleaching (to increase whiteness) (Rouette, 2001). The conventional methods used for these processes are chemical-intensive, nonspecific, and involve the application of high temperatures under alkaline conditions, making them environmentally unfriendly (Bhardwaj et al., 2019). To combat these issues, several research recommendations advocate for the use of more environmentally friendly enzyme-based treatments (Lenting et al., 2002; Agrawal et al., 2004; Lenting and Warmoesken, 2004).

Among the enzymes used in the textile industry, xylanase is involved in the desizing, bioscouring, biostoning of denim garments, fabric softening, releasing extra dye, and biobleaching (Singh, 1999; Polizeli et al., 2005). Recognizing this, several research groups have conducted studies to find cellulase-free xylanase enzymes with high thermal and alkaline pH stability for use in desizing and scouring processes in the textile industry (Bajpai, 2012). Some reports indicate the successful utilization of xylanase extracted from *B. pumilus* and *Bacillus stearothermophilus* SDX for the desizing of cotton and micropoly fabrics and bioscouring (Battan et al., 2012; Saurabh et al., 2008).

### Application of xylanase in food Industry

The biocatalyst xylanase has a wide range of applications in the food industry due to its ethanol tolerance, stability across a broad pH range, thermal stability, salt tolerance, and resistance to various metal ions (Qeshmi et al., 2020).

#### Baking industry

Xylanase has gained immense interest in the baking industry for its role in improving dough attributes and bread quality (Pontonio et al., 2020). Baking industries use wheat flour as a raw material for making bread. Wheat grain contains hemicellulose (arabinoxylan) in water-soluble and water-insoluble forms (Courtin and Delcour, 2002). The water-insoluble fibers in wheat grain hinder the quality of bread (Cavella et al., 2008). In the baking process, xylanase is added to the dough to convert water-insoluble hemicellulose into a water-soluble form, which helps improve the rheological properties of the dough. These improvements include increased volume, more uniform, and finer crumbs, and decreased dough firmness (Butt et al., 2008; Camacho and Aguilar, 2003).

Omar Al-Widyan et al. (2008) reported that xylanases produced from different types of microorganisms significantly improved loaf volume, loaf color, crumb texture, and firmness of the bread. Similarly, Driss et al. (2013) demonstrated that the addition of xylanase decreased the springiness and gumminess of bread. Xylanase also enhances bread quality and extends shelf life by reducing the staling rate (Harris and Ramalingam, 2010).

#### Fruit juice clarification

The presence of polysaccharides such as celluloses, pectins, hemicelluloses, and surface-bound lignin in fruit juice negatively affects its quality, leading to haze, color, and viscosity issues (Danalache et al., 2018). Since the acceptability of cloudy and turbid juices is very low, fruit juice industries use different plant cell wall-degrading enzymes such as pectinase, cellulase, and xylanase to remove these undesired properties (Pinelo et al., 2010; Kumar et al., 2014). Xylanase acts on the hemicellulose content present in freshly squeezed juice, clarifying its excess turbidity and cloudiness (Kaushal et al., 2021).

Nowadays, the utilization of xylanase for the clarification and extraction of fruit juices has gained more attention, with many research outcomes published showing the potential of xylanase for juice extraction and clarification from various sources. The potential of xylanase from *Streptomyces* species was investigated, and the clarification of juices from different fruits, such as orange (20.9%), mousambi (23.6%), and pineapple (27.9%) was reported by Rosmine et al. (2017). Kumar and his research group demonstrated the potential application of immobilized xylanase from *B. pumilus* VLK-1 for the clarification of orange and grape juice, achieving 29% and 26% clarity, respectively (Kumar et al., 2014). Additionally, fruit juices from orange, grape, kiwi, apple, pomegranate, peach, and apricot treated with endo-β-1,4-xylanase extracted from *Pediococcus acidilactici* GC25 showed decreased turbidity (Adiguzel et al., 2019). These research outcomes indicate the potential and applicability of xylanase in the fruit juice industry.

### Application of xylanase in biofuel production

Second-generation biofuels from renewable resources have gained attention as alternatives to fossil fuels because they do not compete with food production and can provide environmental and economic benefits (Viikari et al., 2012; Sharma and Sharma, 2016). Xylanase, along with other hydrolytic enzymes, is used for the depolymerization of carbohydrate polymers (lignocellulosic biomass) to produce biofuels (Beg et al., 2001; Lee, 2009). Research indicates the role of xylanase in the saccharification of lignocellulosic biomass for biofuel production (Ramanjaneyulu et al., 2017; Basit et al., 2018).

**Fig. 3:**
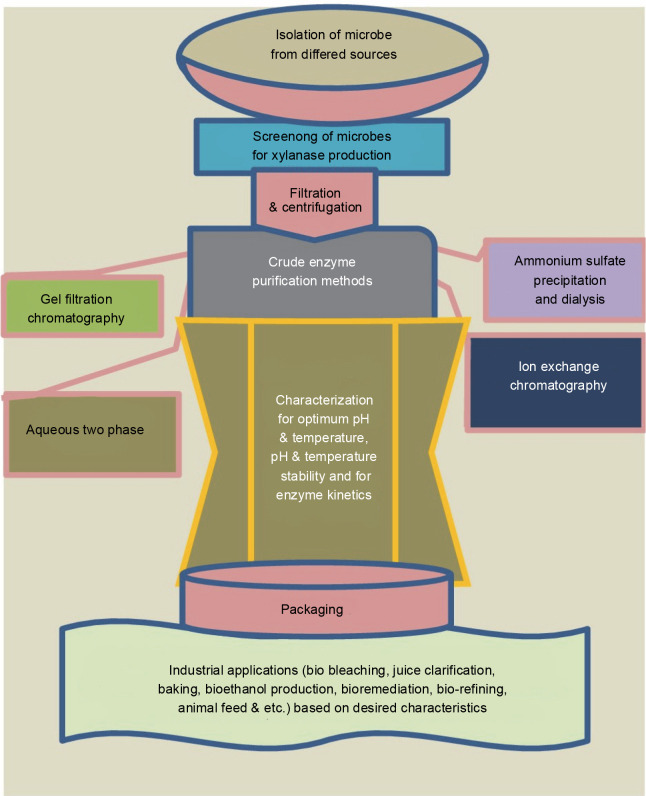
General schematic presentation of xylanase production, purification, characterization, and applications

For the production of biofuels from lignocellulosic biomass, various integrated strategies that improve ethanol production have been developed. These processes include simultaneous saccharification fermentation (SSF), simultaneous saccharification and co-fermentation (SSCF), and consolidated bioprocessing (CB) (Sun and Cheng, 2002; Malhotra and Chapadgaonkar, 2018). Cellulase and xylanase enzymes are used for bioethanol production by saccharification and co-fermentation (SSCF) of anhydrous ammonia-pretreated Napier grass (Yasuda et al., 2014). Song and coworkers demonstrated that using xylanase together with cellulase improved the conversion efficiency of the reducing sugars derived from corn stover (48.5%) and rice straw (31.1%) (Song et al., 2016). Figure 3 illustrates the general schematics of xylanase production, purification, characterization, and applications.

## Future perspectives

Exploring super xylanases with high specific activities and stability under different physicochemical conditions is a crucial future task for researchers and academicians. Isolating extremophile microbial strains that can withstand high or low temperatures and pH from various extreme environments and optimizing their growth conditions for xylanase production is essential. Emerging biotechnological tools such as metagenomics and recombinant DNA technology are used to screen and select the genes responsible for xylanase production and transfer them into expression systems, respectively. Combining synthetic biology (DNA oligosynthesis) and conventional recombinant DNA technology can be used for high xylanase production with desired industrial properties. To protect the enzyme from harsh conditions (high temperature, surfactants, and oxidizing agents) and improve its stability, enzyme immobilization should be carried out.

## Conclusion

Xylanase is an enzyme that randomly splits the β-1,4 linkage of xylan, breaking it down into its constituent components. The most common class of xylanase involved in xylan hydrolysis is endo-1,4-β-xylanase. Xylanases are classified into different glycoside hydrolase (GH) families, with major xylanases from GH10 or GH11 families, and some from GH5, GH7, GH8, and GH43 families. Xylanase has potential applications in various industries, including textile, paper and pulp, food, animal feed, bioremediation, and bio-refinery industries.

For the production of xylanase with desired characteristics suitable for the aforementioned industrial applications, new technologies (synthetic biology and directed evolution) should be combined with conventional methods. To meet industrial process requirements and solve problems related to native xylanase, future research should focus on sequence-based and function-based screening of novel xylanases from extreme environments, genetic engineering, and directed evolution. This approach should be prioritized over conventional methods (culture-dependent screening methods), which can only culture a small fraction of the microbial population using commercially available media.

## References

[cit0001] Adigüzel A.O., Tunçer M, (2016) Production, characterization and application of a xylanase from Streptomyces species AOA40 in fruit juice and bakery industries. Food Biotechnol. 30: 189–218.

[cit0002] Agrawal P.B., Nierstrasz V.A., Warmoeskerken M.M.C.G. (2004) Enhanced bioscouring performance. [in:] Proceedings of the 4th Autex conference. Roubaix, France 2224: 165–173.

[cit0003] Ahmed S., Riaz S., Jamil A. (2009) Molecular cloning of fungal xylanases: an overview. Appl. Microbiol. Biotechnol. 84: 19–35. 19568746 10.1007/s00253-009-2079-4

[cit0004] Al-Widyan O., Khataibeh M.H., Abu-Alruz K. (2008) The use of xylanases from different microbial origin in bread baking and their effects on bread qualities. J. Appl. Sci. 8: 672–676.

[cit0005] Bajpai P. (2012) Environmentally benign approaches for pulp bleaching. [in:] *Environmentally benign approaches for pulp bleaching*. Second Ed. Elsevier.

[cit0006] Bajpai P. (2022) Sources, production, and classification of xylanases. Microb. Xylanol. Enzym. 2022: 69–97. 10.1016/b978-0-323-99636-5.00003-8

[cit0007] Bandikari R., Poondla V., Obulam V.S.R. (2014) Enhanced production of xylanase by solid state fermentation using Trichoderma koeningi isolate: effect of pretreated agro-residues. 3 Biotech. 4: 655–664. 10.1007/s13205-014-0239-4 PMC4235890 28324314

[cit0008] Basit A., Liu J., Miao T., Zheng F., Rahim K., Lou H., Jiang W. (2018) Characterization of two endo-β-1, 4-xylanases from Myceliophthora thermophila and their saccharification efficiencies, synergistic with commercial cellulase. Front. Microbiol. 9: 1–11. 29491860 10.3389/fmicb.2018.00233 PMC5817056

[cit0009] Basit A., Jiang W., Rahim K. (2020) Xylanase and its industrial applications. [in:] *Biotechnological applications of biomass*. Basso T.P., Basso T.O., Basso L.C. (Ed.). Intechopen. London, UK: 638.

[cit0010] Battan B., Dhiman S.S., Ahlawat S., Mahajan R., Sharma J. (2012) Application of thermostable xylanase of Bacillus pumilus in textile processing. Indian J. Microbiol. 52: 222–229. 23729886 10.1007/s12088-011-0118-1 PMC3386437

[cit0011] Baysal Z., Uyar F., Aytekin Ç. (2003) Solid state fermentation for production of a-amylase by a thermotolerant Bacillus subtilis from hot-spring water. Process. Biochem. 38: 1665–1668.

[cit0012] Bedford M.R., Partridge G.G. (2010) *Enzymes in farm animal nutrition*. CABI Publishing: 15–16.

[cit0013] Beg Q.K., Kapoor M., Mahajan L. Hoondal G.S. (2001) Microbial xylanases and their industrial applications: a review. Appl. Microbiol. Biotechnol., 56: 326–338. 11548999 10.1007/s002530100704

[cit0014] Béra-Maillet C., Devillard E., Cezette M., Jouany J.P., Forano E. (2005) Xylanases and carboxymethylcellulases of the rumen protozoa Polyplastron multivesiculatum Eudiplodinium maggii and Entodinium species. FEMS Microbiol. Lett. 244: 149–156. 15727834 10.1016/j.femsle.2005.01.035

[cit0015] Bhardwaj N., Kumar B., Verma P. (2019) A detailed overview of xylanases: an emerging biomolecule for current and future prospective. Bioresour. Bioproces. 6: 1–36.

[cit0016] Bonuglisantos R.C., Vasconcelos M.R.D.S., Passarini M.R.Z., Vieira G.A.L., Lopes V.C.P., Mainardi P.H., Santos J.A.D., Duarte L.D.A., Otero I.V.R., Yoshida A.M.D.S. (2015) Marine-derived fungi: diversity of enzymes and biotechnological applications. Front. Microbiol. 6: 269. 25914680 10.3389/fmicb.2015.00269 PMC4392690

[cit0017] Branco R., Serafim L., Xavier A. (2019) Second generation bioethanol production: on the use of pulp and paper industry wastes as feedstock. Fermentation 5: 4.

[cit0018] Brennan Y., Callen W.N., Christoffersen L., Dupree P., Goubet F., Healey S., et al. (2004) Unusual microbial xylanases from insect guts. Appl. Environ. Microbiol. 70: 3609–3617. 15184164 10.1128/AEM.70.6.3609-3617.2004 PMC427792

[cit0019] Buswell J.A., Chang S.T. (1994) Biomass and extracellular hydrolytic enzyme production by six mushroom species grown on soybean waste. Biotechnol. Lett. 16: 1317–1322.

[cit0020] Butt M.S., Tahir-Nadeem M., Ahmad Z., Sultan M.T. (2008) Xylanases and their applications in baking industry. Food Technol. Biotech. 46: 22–31.

[cit0021] Camacho N.A., Aguilar O.G. (2003) Production, purification, and characterization of a low-molecular-mass xylanase from Aspergillus sp. and its application in baking. Appl. Biochem. Biotech. 104: 159–171. 10.1385/abab:104:3:159 12665668

[cit0022] Carroll A. Somerville C. (2009) Cellulosic biofuels. Annu. Rev. Plant Biol. 60: 165–182. 19014348 10.1146/annurev.arplant.043008.092125

[cit0023] Castanares A., Hay A.J., Gordon A.H., McCrae S.I., Wood, T.M. (1995) d-Xylan-degrading enzyme system from the fungus Phanerochaete chrysosporium. isolation and partial characterisation of an α-(4-O-methyl)-d-glucuronidase. J. Biotech. 43: 183–194. 10.1016/0168-1656(95)00128-x 8590644

[cit0024] Cavella S., Romano A., Giancone T., Masi P. (2008) The influence of dietary fibres on bubble development during bread making. [in:] *Bubbles in food 2*. AACC International Press: 311–321.

[cit0025] Chandra R., Abhishek A., Sankhwar M. (2011) Bacterial decolorization and detoxification of black liquor from rayon grade pulp manufacturing paper industry and detection of their metabolic products. Bioresour. Technol. 102: 6429–6436. 21482463 10.1016/j.biortech.2011.03.048

[cit0026] Collins T., Gerday C., Feller G. (2005) Xylanases, xylanase families and extremophilic xylanases. FEMS Microbiol. Rev. 29: 3–23. 15652973 10.1016/j.femsre.2004.06.005

[cit0027] Collins T., Meuwis M.A., Stals I., Claeyssens M., Feller G., Gerday C. (2002) A novel family 8 xylanase, functional and physicochemical characterization. J. Biol. Chem. 277: 35133–35139. 12089151 10.1074/jbc.M204517200

[cit0028] Corral O.L., Villasenor-Ortega F. (2006) Xylanases. [in:] *Advances in agricultural and food biotechnology. Research signpost*. Guevara-González R.G., Torres-Pacheco I. (Ed.). Kerala, India: 305–322.

[cit0029] Courtin C.M., Delcour J.A. (2002) Arabinoxylans and endoxylanases in wheat flour bread-making. J. Cereal Sci. 35: 225–243.

[cit0030] Danalache F., Mata P., Alves V.D., Moldão-Martins M. (2018) *Enzyme-assisted extraction of fruit juices*. Elsevier Inc., New York.

[cit0031] de Souza H.F., Borges L.A., Gonçalves V.D.D.P., Dos Santos J.V., Bessa M.S., Carosia M.F. et al. (2022) Recent advances in the application of xylanases in the food industry and production by actinobacteria: a review. Food Res. Inter. 162: 112103. 10.1016/j.foodres.2022.112103 36461343

[cit0032] de Souza-Cruz P.B., Freer J., Siika-Aho M., Ferraz A. (2004) Extraction and determination of enzymes produced by Ceriporiopsis subvermispora during biopulping of Pinus taeda wood chips. Enzyme Microbial Technol. 34: 228–234.

[cit0033] Doner L.W., Hicks K.B. (1999) Isolation of hemicellulose from corn fiber by alkaline hydrogen peroxide extraction. Cereal Chem. 74: 176–181.

[cit0034] Driss D., Bhiri F., Siela M., Bessess S., Chaabouni S., Ghorbel R. (2013) Retracted: Improvement of Breadmaking Quality by Xylanase GH11 from Penicillium occitanis Pol6. J. Texture Stud. 44(1): 75–84. 10.1111/j.1745-4603.2012.00367.x 35484802

[cit0035] Garai D., Kumar V. (2013) Aqueous two phase extraction of alkaline fungal xylanase in PEG/phosphate system: optimization by Box–Behnken design approach. Biocatal. Agric. Biotechnol. 2: 125–131.

[cit0036] Garcia-Kirchner O., Munoz-Aguilar M., Pérez-Villalva R., Huitron-Vargas C. (2002) Mixed submerged fermentation with two filamentous fungi for cellulolytic and xylanolytic enzyme production. [in:] *Biotechnology for fuels and chemicals*. Humana Press, Totowa, NJ: 1105-1114. 10.1385/abab:98-100:1-9:1105 12018234

[cit0037] Garg G., Dhiman S.S., Mahajan R., Kaur A., Sharma J. (2011) Bleach-boosting effect of crude xylanase from Bacillus stearothermophilus SDX on wheat straw pulp. New Biotechnol. 28: 58–64. 10.1016/j.nbt.2010.07.020 20709630

[cit0038] Girelli A.M., Astolfi M.L., Scuto F.R. (2020) Agro-industrial wastes as potential carriers for enzyme immobilization: a review. Chemosphere 244: 125368. 31790990 10.1016/j.chemosphere.2019.125368

[cit0039] Gírio F.M., Fonseca C., Carvalheiro F., Duarte L.C., Marques S., Bogel-Lukasik R. (2010) Hemicelluloses for fuel ethanol: a review. Bioresour. Technol. 101: 4775–4800. 20171088 10.1016/j.biortech.2010.01.088

[cit0040] Glyk A., Scheper T., Beutel S. (2015) PEG-salt aqueous two-phase systems: an attractive and versatile liquid–liquid extraction technology for the downstream processing of proteins and enzymes. Appl. Microbiol. Biotechnol. 99: 6599–6616. 26150244 10.1007/s00253-015-6779-7

[cit0041] Goluguri B.R., Thulluri C., Cherupally M., Nidadavolu N., Achuthananda D., Mangamuri L.N., Addepally U. (2012) Potential of thermo and alkali stable xylanases from Thielaviopsis basicola (MTCC-1467) in biobleaching of wood Kraft pulp. Appl. Biochem. Biotechnol. 167: 2369–2380. 22717769 10.1007/s12010-012-9765-x

[cit0042] Gómez García R., Medina Morales M.A., RodrXguez R., Farruggia B.M., Picó G.A., Aguilar C.N. (2018) Production of a xylanase by Trichoderma harzianum (Hypocrea lixii) in solid-state fermentation and its recovery by an aqueous two-phase system. Can. J. Biotechnol. 2: 108–115.

[cit0043] Goswami G.K., Pathak R.R. (2013) Microbial xylanases and their biomedical applications: a review. Int. J. Basic Clin. Pharmacol. 2: 237–246.

[cit0044] Gote M.M, (2004) Isolation, purification and characterization of thermostable a-galactosidase from Bacillus stearothermophilus (NCIM-5146). Global Biogeochem. Cycles 23: 798–802.

[cit0045] Harris A.D., Ramalingam C. (2010) Xylanases and its application in food industry: a review. J. Exp. Sci., 1: 1–11.

[cit0046] Hatakka A., Hammel K.E. (2010) Fungal biodegradation of lignocelluloses. [in:] *Mycota X. Industrial Applications*, 2nd edn. Hofrichter M., Ullrich R. (Eds.). Berlin, Heidelberg: Springer.

[cit0047] Ho H.L., Jamila S. (2014) Optimisation of medium formulation and growth conditions for xylanase production by Aspergillus brasiliensis in submerged fermentation (SmF). J. Biodivers. Biopros. Dev. 1: 102.

[cit0048] Horikoshi K., Atsukawa Y. (1973) Xylanase produced by alkalophilic bacillus No. C-59-2. Agric. Biol. Chem. 37: 2097–2103.

[cit0049] Howard R.L., Abotsi E., Jansen E.L., Howard S. (2003) Lignocellulose biotechnology: issues of bioconversion and enzyme production. Afr. J. Biotechnol. 2: 602–619.

[cit0050] Hunt C.J., Tanksale A., Haritos V.S. (2016) Biochemical characterization of a halotolerant feruloyl esterase from Actinomyces spp.: refolding and activity following thermal deactivation. Appl. Microbiol. Biotechnol. 100: 1777–1787. 26497017 10.1007/s00253-015-7044-9

[cit0051] Iqbal M., Tao Y., Xie S., Zhu Y., Chen D., Wang X., Huang L., Peng D., Sattar A., Shabbir M.A.B., Hussain H.I. (2016) Aqueous two-phase system (ATPS): an overview and advances in its applications. Biol. Proc. Online 18: 1–18. 10.1186/s12575-016-0048-8 PMC5084470 27807400

[cit0052] Irfan M., Syed Q. (2012) Partial purification and characterization of Xylanase from Trichoderma viride produced under SSF. Int. J. Appl. Res. Nat. Prod. 5: 7–11.

[cit0053] Jain A., Morlok C.K., Henson J.M. (2013) Comparison of solid-state and submerged-state fermentation for the bioprocessing of switchgrass to ethanol and acetate by Clostridium phytofermentans. Appl. Microbiol. Biotechnol. 97: 905–917. 23111595 10.1007/s00253-012-4511-4

[cit0054] Jensen J.K., Busse Wicher M., Poulsen C.P., Fangel J.U., Smith P.J., Yang J.Y., et al. (2018) Identification of an algal xylan synthase indicates that there is functional orthology between algal and plant cell wall biosynthesis. New Phytologist. 218: 1049–1060. 29460505 10.1111/nph.15050 PMC5902652

[cit0055] Kamble R.D., Jadhav A.R. (2012) Isolation, purification and characterization of xylanase produced by a new species of Bacillus in solid state fermentation. Inter. J. Microbiol. 1: 1–8. 10.1155/2012/683193 PMC3270423 22315613

[cit0056] Kaushal J., Khatri M., Singh G., Arya S.K. (2021) A multifaceted enzyme conspicuous in fruit juice clarification: an elaborate review on xylanase. Inter. J. Biol. Macromol. 193: 1350–1361. 10.1016/j.ijbiomac.2021.10.194 34740694

[cit0057] Khanahmadi M., Mitchell D.A., Beheshti M., Roostaazad R., Sánchez L.R. (2006) Continuous solid-state fermentation as affected by substrate flow pattern. Chem. Eng. Sci. 61: 2675–2687.

[cit0058] Knob A., Terrasan C., Carmona E. (2010) β-Xylosidases from filamentous fungi: an overview. World J. Microbiol. Biotechnol. 26: 389–407.

[cit0059] Ko C.H., Lin Z.P., Tu J., Tsai C.H., Liu C.C., Chen H.T., Wang T.P. (2010) Xylanase production by Paenibacillus campinasensis BL11 and its pretreatment of hardwood kraft pulp bleaching. Inter. Biodeterior. Biodegrad. 64: 13–19.

[cit0060] Kumar L., Nagar S., Mittal A., Garg N., Gupta V.K. (2014) Immobilization of xylanase purified from Bacillus pumilus VLK-1 and its application in enrichment of orange and grape juices. J. Food Sci. Technol. 51: 1737–1749. 25190829 10.1007/s13197-014-1268-z PMC4152508

[cit0061] Lee J.W., Park J.Y., Kwon M., Cho I.G. (2009) Purification and characterization of a thermostable xylanase fromnthe brown rot fungus Laetiporus sulphurous. J. Biosci. Bioeng. 107: 33–37. 19147106 10.1016/j.jbiosc.2008.09.006

[cit0062] Lenting H.B.M., Warmoeskerken M.M.C.G. (2004) A fast, continuous enzyme-based pretreatment process concept for cotton containing textiles. Biocatal. Biotransform. 22: 361–368.

[cit0063] Lenting H.B.M., Zwier E., Nierstrasz V.A. (2002) Identifying important parameters for a continuous bioscouring process. Text Res. J. 72: 825–831.

[cit0064] Li X., She Y., Sun B., Song H., Zhu Y., Lv Y., Song H. (2010) Purification and characterization of a cellulase-free, thermostable xylanase from Streptomyces rameus L2001 and its biobleaching effect on wheat straw pulp. Biochem. Eng. J. 52: 71–78.

[cit0065] Li X., Dilokpimol A., Kabel M.A., de Vries R.P. (2022) Fungal xylanolytic enzymes: diversity and applications. Bioresour. Technol. 344: 126290. 34748977 10.1016/j.biortech.2021.126290

[cit0066] Liu J., Willför S., Xu C. (2015) A review of bioactive plant polysaccharides: biological activities, functionalization, and biomedical applications. Bioact. Carbohydr. Diet Fibre 5: 31–61.

[cit0067] Liu X., Kokare C.B. (2017) Chapter 11: Microbial enzymes of use in industry. [in:] *Biotechnology of microbial enzymes.* Brahmachari G. (Ed). Academic Press, Cambridge: 267–298.

[cit0068] Lopez G., Estrada P. (2014) Effect of temperature on xylanase II from Trichoderma reesei QM 9414: a calorimetric, catalytic, and conformational study. Enzyme Res. 2014: 16. 10.1155/2014/708676 PMC4170777 25276420

[cit0069] Malhotra G., Chapadgaonkar S.S. (2018) Production and applications of xylanases an overview. BioTechnologia 99: 59–72.

[cit0070] Mathur S., Kumar S., Rao N.J. (2005) Action of xylanase pre-bleaching on wheat straw and oxygen delignified wheat straw soda pulps-probable mechanisms. [in:] 59 th Appita Annual Conference and Exhibition: incorporating the 13 th ISWFPC (International Symposium on Wood, Fibre and Pulping Chemistry) Proceedings. Auckland, New Zealand: 631.

[cit0071] Mirande C., Mosoni P., Bera-Maillet C., Bernalier-Donadille A., Forano E. (2010) Characterization of Xyn10A, a highly active xylanase from the human gut bacterium Bacteroides xylanisolvens XB1A. Appl. Microbiol. Biotechnol. 87: 2097–2105. 20532756 10.1007/s00253-010-2694-0

[cit0072] Mitreva-Dautova M., Roze E., Overmars H., De Graaff L., Schots A., Helder J., et al. (2006) A symbiont-independent endo-1,4-β-xylanase from the plant-parasitic nematode Meloidogyne incognita. Mol. Plant Microbe Interact. 19(5): 521–529. 16673939 10.1094/MPMI-19-0521

[cit0073] Motta F.L., Andrade C.C.P., Santana M.H.A. (2013) A review of xylanase production by the fermentation of xylan: classification, characterization and applications. Sustainable degradation of lignocellulosic biomass-techniques, applications and commercialization. [in:] *TechOpen*. NewYork: 251–275.

[cit0074] Nagar S., Jain R.K., Thakur V.V., Gupta V.K. (2013) Biobleaching application of cellulase poor and alkali stable xyla-nase from Bacillus pumilus SV-85S. 3 Biotech. 3: 277–285. 10.1007/s13205-012-0096-y PMC3723860 28324585

[cit0075] Nair S.G., Shashidhar S. (2008) Fungal xylanase production under solid state and submerged fermentation conditions. Afri. J. Microbiol. Res. 2: 82–86.

[cit0076] Ng H.S., Chai C.X.Y., Chow Y.H., Loh W.L.C., Yim H.S., Tan J.S., Lan, J.C.W. (2018) Direct recovery of Bacillus subtilis xylanase from fermentation broth with an alcohol/salt aqueous biphasic system. J. Biosci. Bioengin. 125: 585–589. 10.1016/j.jbiosc.2017.12.010 29339003

[cit0077] Paloheimo M., Piironen J., Vehmaanperä J. (2010) Xylanases and cellulases as feed additives. [in:] *Enzymes in farm animal nutrition*. CABI Publishing: 12–53. 10.1079/9781845936747.0012

[cit0078] Pandey A., Selvakumar P., Soccol C.R., Nigam P. (1999) Solid state fermentation for the production of industrial enzymes. Curr. Sci. 77: 149–162.

[cit0079] Pandey A., Negi S., Binod P., Larroche C. (2014) *Pretreatment of biomass: processes and technologies*. Elsevier.

[cit0080] Pandey A., Soccol C.R., Mitchell D. (2000) New developments in solid state fermentation. [in:] *Bioprocess and products*. Process Biochem. 35: 153–169.

[cit0081] Pandya J.J., Gupte A. (2012) Production of xylanase under solid-state fermentation by Aspergillus tubingensis JP-1 and its application. Bioprocess Biosyst. Eng. 35: 769–779. 22271252 10.1007/s00449-011-0657-1

[cit0082] Pasalari A., Homaei A. (2022) Isolation and molecular identification of xylanase-producing bacteria from Ulva flexuosa of the Persian Gulf. Processes 10: 1834.

[cit0083] Passos A.A., Park I., Ferket P., von Heimendahl E., Kim S.W. (2015) Effect of dietary supplementation of xylanase on apparent ileal digestibility of nutrients, viscosity of digesta, and intestinal morphology of growing pigs fed corn and soybean meal based diet. Animal Nutr. 1: 19–23. 10.1016/j.aninu.2015.02.006 PMC5884468 29766982

[cit0084] Pei S., van De Lindt J.W., Popovski M., Berman J.W., Dolan J.D., Ricles J., et al. (2016) Cross-laminated timber for seismic regions: progress and challenges for research and implementation. J. Struct. Eng. 142: E2514001.

[cit0085] Peng F., Peng P., Xu F., Sun R.C. (2012) Fractional purification and bioconversion of hemicellulose. Biotechnol. Adv. 30: 879–903. 22306329 10.1016/j.biotechadv.2012.01.018

[cit0086] Pinelo M., Zeuner B., Meyer A.S. (2010) Juice clari cation by protease and pectinase treatments indicates new roles of pectin and protein in cherry juice turbidity. Food Bioprod. Process. 88: 259–265.

[cit0087] Pinheiro V.P., Ferreira J.A., Betini J.H.A., Kamimura E., Polizeli S., M.L.T.M. (2021) Utilizing a novel fungal enzymatic cocktail as an eco-friendly alternative for cellulose pulp biobleaching. BioResources 16: 7509–7529.

[cit0088] Pontonio E., Dingeo C., Di Cagno R., Blandino M., Gobbetti M., Rizzello C.G. (2020) Brans from hull-less barley, emmer and pigmented wheat varieties: from by-products to bread nutritional improvers using selected lactic acid bacteria and xylanase. Inter. J. Food Microbiol. 313: 108384. 10.1016/j.ijfoodmicro.2019.108384 31670259

[cit0089] Prasad M., Sethi R. (2013) Screening for xylanase producing microorganisms from marine sources. Int. J. Microbiol. Appl. Sci. 2: 489–492.

[cit0090] Qeshmi F.I., Homaei A., Fernandes P., Hemmati R., Dijkstra B.W., Khajeh K. (2020) Xylanases from marine microorganisms: a brief overview on scope, sources, features and potential applications. Biochim. Biophys. Acta 1868: 140312. 10.1016/j.bbapap.2019.140312 31740412

[cit0091] Raj A., Kumar S., Singh S.K., Prakash J. (2018) Production and purification of xylanase from alkaliphilic Bacillus licheniformis and its pretreatment of eucalyptus kraft pulp. Biocatal. Agric. Biotechnol. 15: 199–209.

[cit0092] Ramakrishnan V., Goveas L.C., Suralikerimath N., Jampani C., Halami P.M., Narayan B. (2016) Extraction and purification of lipase from Enterococcus faecium MTCC5695 by PEG/phosphate aqueous-two phase system (ATPS) and its biochemical characterization. Biocatal. Agric. Biotechnol. 6: 19–27.

[cit0093] Rosmine E., Sainjan N.C., Silvester R., Alikkunju A., Varghese S.A. (2017) Statistical optimisation of xylanase production by estuarine Streptomyces sp. and its application in clarification of fruit juice. J. Genet. Eng. Biotechnol. 15: 393–401. 30647677 10.1016/j.jgeb.2017.06.001 PMC6296605

[cit0094] Rouette H.K. (2001) *Encyclopedia of textile nishing*. Springer, Berlin: 1–3.

[cit0095] Roy S., Durra T., Sarkar T.S., Ghosh S. (2013) Novel xylanase from Simplicillium obclavatum 9604: Comparative analysis of production, purification and characterization of enzymes from submerged and solid state fermentation. SpringerPlus 2: 1–10. 24010040 10.1186/2193-1801-2-382 PMC3755804

[cit0096] Saurabh S.D., Jitender S., Bindu B. (2008) Pretreatment processing of fabrics by alkalothermophilic xylanase from Bacillus stearothermophilus SDX. Enzyme Microb. Technol. 43: 262–269.

[cit0097] Sharma A., Thakur V.V., Shrivastava A., Jain R.K., Mathur R.M., Gupta R., Kuhad R.C. (2014) Xylanase and laccase based enzymatic kraft pulp bleaching reduces adsorbable organic halogen (AOX) in bleach effluents: a pilot scale study. Bioresour. Technol. 169: 96–102. 25036336 10.1016/j.biortech.2014.06.066

[cit0098] Sharma N., Sharma N. (2016) Bioethanol production from alkaline hydrogen peroxide pretreated Populus deltoids wood using hydrolytic enzymes of Bacillus stratosphericus N12 (M) and Bacillus altitudinis Kd1 (M) under different modes of separate hydrolysis and fermentation by monoculture and co-culture culture combinations of ethanologens. Int. J. Bioassays 5: 4810–4816.

[cit0099] Silva L.A.O., Fanchini Terrasanb C.R., Cano Carmona E. (2015) Purification and characterization of xylanases from Trichoderma inhamatum. Electr. J. Biotechnol. 18: 307–313.

[cit0100] Singh S., Dutt D., Tyagi C.H. (2013) Screening of xylanases from indigenously isolated white rot fungal strains for possible application in pulp biobleaching. Open Access Sci. Rep. 2: 262.

[cit0101] Singh S., Sidhu G.K., Kumar V., Dhanjal D.S., Datta S., Singh J. (2019) Fungal xylanases: sources, types, and biotechnological applications. [in:] *Recent advancement in white biotechnology through fungi*. Springer: 405–428.

[cit0102] Singhania R.R., Patel A.K., Soccol C.R., Pandey A. (2009) Recent advances in solid-state fermentation. Biochem. Eng. J. 44: 13–18.

[cit0103] Sizova M.V., Izquierdo J.A., Panikov N.S., Lynd L.R. (2011) Cellulose-and xylan degrading thermophilic anaerobic bacteria from biocompost. Appl. Environ. Microbiol. 77: 2282–2291. 21317267 10.1128/AEM.01219-10 PMC3067422

[cit0104] Song H.T., Gao Y., Yang Y.M., Xiao W.J., Liu S.H., Xia W.C., et al. (2016) Synergistic effect of cellulase and xylanase during hydrolysis of natural lignocellulosic substrates. Bioresour. Technol. 219: 710–715. 27560367 10.1016/j.biortech.2016.08.035

[cit0105] Sridevi A., Ramanjaneyulu G., Suvarnalatha Devi P. (2017) Biobleaching of paper pulp with xylanase produced by Trichoderma asperellum. 3 Biotech. 7: 266. 10.1007/s13205-017-0898-z PMC5533674 28794921

[cit0106] Subramaniyam R., Vimala R. (2012) Solid state and submerged fermentation for the production of bioactive substances: a comparative study. Int. J. Sci. Nat. 3: 480–486.

[cit0107] Sun Y., Cheng J., (2002) Hydrolysis of lignocellulosic materials for ethanol production: a review. Bioresour. Technol. 83: 1–11. 12058826 10.1016/s0960-8524(01)00212-7

[cit0108] Taibi Z., Saoudi B., Boudelaa M., Trigui H., Belghith H., Gargouri A., Ladjama A. (2012) Purification and biochemical characterization of a highly thermostable xylanase from Actinomadura sp. strain Cpt20 isolated from poultry compost. Appl. Biochem. Biotech. 166: 663–679. 10.1007/s12010-011-9457-y 22161140

[cit0109] Tarasov D., Leitch M., Fatehi P. (2018) Lignin – carbohydrate complexes: properties, applications, analyses, and methods of extraction. A review. Biotechnol. Biofuel. 11: 269. 10.1186/s13068-018-1262-1 PMC6162904 30288174

[cit0110] Thomas L., Joseph A., Singhania R.R., Patel A.K., Pandey A. (2017) Industrial enzymes: xylanases. [in:] *Current developments in biotechnology and bioengineering*. Elsevier: 127–148.

[cit0111] Verma D., Satyanarayana T. (2020) Xylanolytic extremozymes retrieved from environmental metagenomes: characteristics, genetic engineering, and applications. Front. Microbiol. 11: 551109. 33042057 10.3389/fmicb.2020.551109 PMC7527525

[cit0112] Viikari L., Vehmaanperä J., Koivula A. (2012) Lignocellulosic ethanol: from science to industry. Biomass Bioenergy 46: 13–24.

[cit0113] Vinkx C.J.A., Delcour J.A. (1996) Rye (Secale cereale L.) arabinoxylans: a critical review. J. Cereal Sci. 24: 1–14.

[cit0114] Wainø M., Ingvorsen K. (2003) Production of β-xylanase and β-xylosidase by the extremely halophilic archaeon Halorhabd usutahensis. Extremophiles 7: 87–93. 12664260 10.1007/s00792-002-0299-y

[cit0115] Walia A., Mehta P., Chauhan A., Kulshrestha S., Shirkot C.K. (2014) Purification and characterization of cellulase-free low molecular weight endo ß-1,4 xylanase from an alkalophilic Cellulosimicrobium cellulans CKMX1 isolated from mushroom compost. World J. Microbiol. Biotechnol. 30: 2597–2608. 24908422 10.1007/s11274-014-1683-3

[cit0116] Xavier A.M., Correia M.F., Pereira S.R., Evtuguin D.V. (2010) Secondgeneration bioethanol from eucalypt sulphite spent liquor. Bioresour. Technol. 101: 2755–2761. 20045313 10.1016/j.biortech.2009.11.092

[cit0117] Yadav M.P., Johnston D.B., Hicks K.B. (2009) Corn fiber gum: new structure/function relationships for this potential beverage flavor stabilizer. Food Hydrocol. 23: 1488–1493.

[cit0118] Yamaura I., Koga T., Matsumoto T., Kato T. (1997) Purification and some properties of endo-l,4-β-d-xylanase from a fresh-water mollusc, Pomacea insularus (de Ordigny). Biosci. Biotechnol. Biochem. 61: 615–620. 9145520 10.1271/bbb.61.615

[cit0119] Yang S., Yang B., Duan C., Fuller D.A., Wang X., Chowdhury S.P., Stavik J., Zhang H., Ni Y. (2019) Applications of enzymatic technologies to the production of high-quality dissolving pulp: a review. Bioresour. Technol. 281: 440–448. 30876797 10.1016/j.biortech.2019.02.132

[cit0120] Yasuda M., Nagai H., Takeo K., Ishii Y., Ohta K. (2014) Bioethanol production through simultaneous saccharification and co-fermentation (SSCF) of low-moisture anhydrous ammonia (LMAA)-pretreated napiegrass (Pennisetum purpureum Schumach). SpringerPlus 3: 333. 26034662 10.1186/2193-1801-3-333 PMC4447740

[cit0121] Zhang L., Xu J., Lei L., Jiang Y., Gao F., Zhou G.H. (2014) Effects of xylanase supplementation on growth performance, nutrient digestibility and non-starch polysaccharide degradation in different sections of the gastrointestinal tract of broilers fed wheat-based diets. Asian-Australas J. Anim. Sci. 27: 855–861. 25050024 10.5713/ajas.2014.14006 PMC4093178

[cit0122] Zhu H., Luo W., Ciesielski P.N., Fang Z., Zhu J.Y., Henriksson G., Himmel M.E., Hu L. (2016) Wood-derived materials for green electronics, biological devices, and energy applications. Chem. Rev. 116: 9305–9374. 27459699 10.1021/acs.chemrev.6b00225

